# Mitigation of *Salmonella* on Food Contact Surfaces by Using Organic Acid Mixtures Containing 2-Hydroxy-4-(methylthio) Butanoic Acid (HMTBa)

**DOI:** 10.3390/foods12040874

**Published:** 2023-02-18

**Authors:** Aiswariya Deliephan, Janak Dhakal, Bhadriraju Subramanyam, Charles G. Aldrich

**Affiliations:** 1 Department of Grain Science and Industry, Kansas State University, 201 Shellenberger Hall, 1301 Mid Campus Drive North, Manhattan, KS 66506-2201, USA; 2 Department of Food Science & Technology, University of Nebraska-Lincoln, 1901 North 21 Street, Lincoln, NE 68588-6205, USA

**Keywords:** food contact surfaces, stainless steel, rubber, plastic, antimicrobial, HMTBa, *Salmonella*

## Abstract

Contaminated surfaces can transmit pathogens to food in industrial and domestic food-handling environments. Exposure to pathogens on food contact surfaces may take place via the cross-contamination of pathogens during postprocessing activities. Formaldehyde-based commercial sanitizers in recent years are less commonly being used within food manufacturing facilities due to consumer perception and labeling concerns. There is interest in investigating clean-label, food-safe components for use on food contact surfaces to mitigate contamination from pathogenic bacteria, including *Salmonella*. In this study, the antimicrobial effects of two types of organic acid mixtures containing 2-hydroxy-4-(methylthio) butanoic acid (HMTBa), Activate DA™ and Activate US WD-MAX™, against *Salmonella* when applied onto various food contact surfaces were evaluated. The efficacy of Activate DA (HMTBa + fumaric acid + benzoic acid) at 1% and 2% and Activate US WD-MAX (HMTBa + lactic acid + phosphoric acid) at 0.5% and 1% against *Salmonella enterica* (serovars Enteritidis, Heidelberg, and Typhimurium) were evaluated on six different material surfaces: plastic (bucket elevator and tote bag), rubber (bucket elevator belt and automobile tire), stainless steel, and concrete. There was a significant difference in the *Salmonella* log reduction on the material surfaces due to the organic acid treatments when compared to the untreated surfaces. The type of material surface also had an effect on the log reductions obtained. Stainless steel and plastic (tote) had the highest *Salmonella* log reductions (3–3.5 logs), while plastic (bucket elevator) and rubber (tire) had the lowest log reductions (1–1.7 logs) after treatment with Activate US WD-MAX. For Activate DA, the lowest log reductions (~1.6 logs) were observed for plastic (bucket elevator) and rubber (tire), and the highest reductions were observed for plastic (tote), stainless steel, and concrete (2.8–3.2 logs). Overall, the results suggested that Activate DA at 2% and Activate US WD-MAX at 1% are potentially effective at reducing *Salmonella* counts on food contact surfaces by 1.6–3.5 logs.

## 1. Introduction

Foodborne pathogens cause millions of cases of sporadic illness and chronic complications, as well as large and challenging outbreaks in many countries. Surveys estimate that in the United States alone, bacterial enteric pathogens cause 9.4 million episodes of foodborne illness in humans, 55,961 hospitalizations, and 1351 deaths each year [1]. Salmonellosis caused by *Salmonella* Enteritidis is one of the most common foodborne diseases worldwide, accounting for around 93.8 million foodborne illnesses and 155,000 deaths per year worldwide [2]. Reports in the US account for more than one million people being sickened by *Salmonella* each year [1]. In the last decades, regulations have moved in the direction of managing pet food safety at standards equivalent to human food. For instance, under the US Food, Drug, and Cosmetic Act, pet food should be “safe to eat, produced under sanitary conditions, contain no harmful substances, and be truthfully labeled” [3], and pet food contaminated with *Salmonella* is considered adulterated [4]. Facilities producing animal feed, including pet food, must register as human food facilities [5].

Contaminated surfaces can transmit pathogens to food in industrial and domestic food-handling environments. Exposure to pathogens on surfaces may take place either directly via contact with contaminated objects or indirectly via aerosols originating from the surface. Various bacteria of public health significance, including *Salmonella*, *E. coli*, and *Listeria* can survive on hands, sponges, clothes, utensils, and currency for hours or days [6]. Pathogenic bacteria may remain on equipment surfaces even after disinfection procedures are applied, increasing the risks associated with the transmission of diseases [7]. Not only bacteria but mold spores from the environment may also remain on equipment surfaces, on the floor of food manufacturing facilities, on automobile tires, etc., and eventually find their way to contaminating foods. Therefore, domestic and industrial food-handling environments can be important sources of foodborne pathogens and spoilage organisms, including bacteria and molds.

In a pet food manufacturing facility, batch-to-batch sequencing is used as a control measure to reduce hazard contamination [8]. Though effective at reducing chemical hazards, it is found to be ineffective at reducing biological hazards that can occur through the contaminated organic residue and dust remaining on the surfaces of equipment and conveyors, as well as relatively large quantities of food products remaining in the boot of bucket elevator conveyors. In these instances, more strenuous physical cleaning may be necessary [9]. However, the applicability of this option is limited because some of the equipment in pet food manufacturing facilities may not be designed or inaccessible for cleaning. Further complicating biological hazard control on these surfaces is the potential formation of biofilms. *Salmonella* have been shown to maintain a presence on dry surfaces for up to 4 weeks through a biofilm [10]. Preventive measures utilized by the food industry have included the coating of surfaces to limit the establishment of vegetative cells or biofilms [11]. Huss et al. [9] and Schumacher [12] demonstrated that the liquid decontamination of animal food manufacturing equipment that appears effective were not very practical or easy to implement. Generally, a water activity (a_w_) level of 0.87 is required for the growth of most bacterial pathogens of concern, including *Salmonella*, so introducing a water-based sanitizer may raise the a_w_ to levels that allow for *Salmonella* growth. Hypochlorite and sodium chlorite can be effective detergents and sanitizers and are known to penetrate the biofilms developed by *Salmonella*. Because of their potential impact on human health, chloride, and its derivatives, must be rinsed from surfaces prior to manufacturing food for consumption by pet animals or humans. Formaldehyde-based sanitizers also have bactericidal and fungicidal properties and are commonly used in animal production and healthcare facilities. However, in recent years, they have not been commonly used within food manufacturing facilities due to consumer perceptions and labeling concerns and the additional potential carcinogenic effects to employees if handled improperly. Therefore, there is growing interest in clean-label, food-safe components for use on food contact surfaces that mitigate contamination from bacteria like *Salmonella*.

**Figure 1:**
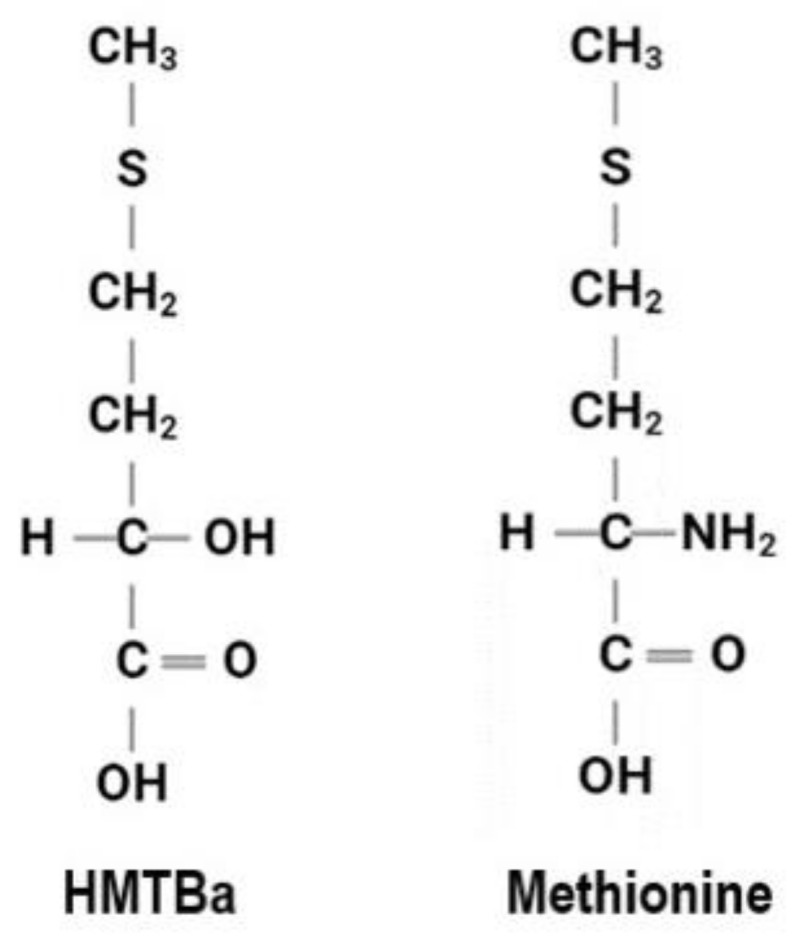
Comparison of the chemical structures of HMTBa (methionine hydroxy analog) and amino acid methionine.

Supplementing animal feed rations with a ‘methionine hydroxy analog’ is an economical way to supply methionine, a limiting amino acid in ruminants. HMTBa (2-hyroxy 4-(methylthio) butanoic acid) is an organic acid and a methionine hydroxy analog. It has been used as a methionine precursor in animal feed due to its unique chemical structure (Figure 1) that allows for protection from some of the microbial degradation of amino acids in the rumen gut. HMTBa also provides the acidifying effects of organic acids. These acidifying effects subsequently provide gut health advantages to the animal by mitigating pathogen growth in the gut [13,14]. A methionine hydroxy analog has also been shown to reduce nitrogen excretion [15], support animal performance during heat-stress [16,17], and offer an antioxidant capacity [18,19,20,21]. Other than these health benefits and its use as a methionine precursor in animal feed supplements, its potential role in inhibiting microbial growth outside of the animal body, like on food contact surfaces, has not been investigated.

Research conducted by the CCL Institute in the Netherlands [22] using HMTBa demonstrated its effectiveness in reducing bacterial populations such as *Salmonella*, *E. coli,* and *Campylobacter*, all of which were found in the drinking water of poultry. HMTBa is one of the main components of Activate DA™ and Activate US WD-MAX™, which are proprietary blends of organic acids from Novus International, Inc., the sponsor of this study. The combination of organic acids effectively reduces the pH of the gastrointestinal tract, promotes the establishment of desirable and more balanced intestinal flora, and aids in digestion, providing more nutrients from feed and improving the performance of the animal. Activate DA (HMTBa + fumaric acid + benzoic acid + silica + mineral oil) is a granular mixture applied to premixes and finished feeds. Activate US WD-MAX (HMTBa + lactic acid + phosphoric acid) is used for the acidification of drinking water for poultry, making the drinking water a less favorable environment for pathogen growth, and it is shown to play an important role in the destruction of harmful micro-organisms in the gut that could affect the birds’ performance. There is limited knowledge on the application of these organic acid mixtures on food contact surfaces as antimicrobial agents to enhance food safety.

The objective of this study was to determine the efficacy of organic acid mixtures Activate DA and Activate US WD-MAX on food contact surfaces on the survival of *Salmonella*.

## 2. Materials and Methods

### 2.1. Organic Acid Mixtures

The organic acid mixtures evaluated in this study were Activate DA (dry formula) and Activate US WD-MAX (liquid formula), which were provided by the study sponsor Novus International (St. Charles, MO, USA). Activate DA is a mixture of HMTBa, fumaric acid, benzoic acid, silica, and mineral oil. Activate US WD-MAX is a mixture of HMTBa, lactic acid, and phosphoric acid. Activate DA, which is in granular form, was further ground to reduce its particle size using a laboratory hammer mill (Verder Scientific, Inc., Newtown, PA, USA) for use in the study.

### 2.2. Inoculum Preparation

*Salmonella enterica* serovars Enteritidis (ATCC 4931), Heidelberg (ATCC 8326), and Typhimurium (ATCC 14028) were procured from the American Type Culture Collection (ATCC, Manassas, VA, USA) and were maintained in tryptic soy broth (TSB)-glycerol (7:3) at −80 °C. Before use, the frozen cultures were streaked onto tryptic soy agar (TSA; BD Difco, Sparks, MD, USA) plates and incubated at 37 °C for 24 h. A single colony of each *Salmonella* strain was inoculated into 10 mL of TSB (BD Difco, Sparks, MD, USA) and incubated at 37 °C for 18 to 24 h. The cultures of each Salmonella serotype thus obtained were centrifuged for 10 min at 5000× *g* (Thermo Scientific, Waltham, MA, USA) at room temperature. The pellets were resuspended in 0.1% presterilized peptone water (BD Difco, Sparks, MD, USA), and an equal volume of each serotype was mixed to obtain the cocktail (∼8 log CFU/mL).

### 2.3. Food Contact Surface Materials and Inoculation

The following food contact surface materials were used in this study: (i) plastic from a bucket elevator, (ii) rubber from a bucket elevator belt, (iii) rubber from an automobile tire, (iv) plastic from a polyethylene tote bag, (v) stainless steel, and (vi) concrete (Figures S1–S6). The plastic from the elevator bucket, the rubber from the elevator belt, and the stainless-steel surface materials were procured from used equipment from the Kansas State University Hal Ross flour mill. The concrete surfaces were made by using poured Quikrete™ (Quikrete Co., Atlanta, GA, USA) in silicone molds, and the rubber from automobile tires was collected from used tires from a local automotive shop. Plastic from industrial tote bags was procured from Sterilite Inc. (Townsend, MA, USA). The materials were cut into square pieces, each measuring 4 × 4 cm.

Prior to the inoculation of the contact surfaces, each surface was soaked in 10% hypochlorite (bleach) solution for 15 min, followed by washing with detergent and rinsing thoroughly. After this, they were immersed in ethanol for 15 min, followed by rinsing with sterile water and drying at 37 °C. The surfaces were all transferred to sterile Petri plates for inoculation with *Salmonella* and subsequent treatment with organic acid mixtures. For inoculation, 1 mL of *Salmonella* inoculum was spot applied to each material surface using a pipette, with the inoculum covering the surface. It was then allowed to dry overnight at 37 °C. To the inoculated surfaces, 1 mL of each treatment (1% or 2% of Activate DA, or 0.5% or 1% of Activate US WD-MAX) was applied and allowed to sit for 15 min. The concentrations of Activate DA and Activate US WD-MAX used for evaluation in this study were based on their minimum inhibitory concentrations (MICs) and minimum bactericidal concentrations (MBCs) against *Salmonella,* as determined in a previous study [23]. For the untreated, each material surface was treated with sterile water instead of the organic acids. For the positive control, the material surfaces were treated with 30% formaldehyde solution, which is the concentration of formaldehyde known to kill *Salmonella* and commonly used in commercial sanitizers like SalCurb™ (Kemin Inc., Des Moines, IA, USA). Following treatment, the surfaces were vortexed in 9 mL of buffered peptone water for 5 min for the recovery of the bacteria and then serially diluted in 0.1% peptone water and plated on xylose lysine deoxycholate (XLD) agar. The plates were incubated at 37 °C for 24 h, and colonies were counted and expressed as log CFU/cm^2^. All surface inoculations and subsequent treatments and enumeration were repeated for a total of 3 replicates.

### 2.4. Confirmative Test for Salmonella

Confirmative test for *Salmonella* for the experiments mentioned above was conducted according to FDA-BAM method (Bacteriological Analytical Manual). In short, buffered peptone water from the pre-enrichment of each treatment sample, 1.0 mL and 0.1 mL, was transferred to 10 mL of Rappaport-Vassiliadis (RV; BD Difco, Sparks, MD, USA) and tetrathionate (TT; BD Difco, Sparks, MD, USA) broths, respectively, and incubated at 42 °C for 24 h for the selective enrichment of *Salmonella*. From each RV and TT broth tube, one loopful was streaked onto xylose lysine deoxycholate (XLD) agar plates in duplicate. Inverted plates were incubated at 37 °C for 24 h. Presumptive positive *Salmonella* colonies appeared as pink colonies with or without black centers, with most positive *Salmonella*-producing colonies having large, glossy black centers or being almost completely black. Presumptive *Salmonella*-positive colonies from XLD plates were then inoculated into triple sugar iron agar (TSI; BD Difco, Sparks, MD, USA) slants by streaking the slant and stabbing the butt and the lysine iron agar (LIA; BD Difco, Sparks, MD, USA) slants by stabbing the butt twice and then streaking the slant. The TSI and LIA slants were incubated at 37 °C for 24 h. The presumptive *Salmonella*-positive TSI reactions had alkaline (red) slants and acid (yellow) butts, while the LIA reactions had an alkaline (purple) butt with acidic (yellow) reaction negative for *Salmonella*. All cultures with an alkaline butt in LIA, regardless of TSI reaction, were retained as potential *Salmonella* isolates. Presumed positive TSI and LIA slant cultures were inoculated into TSB and incubated at 37 °C for 24 h, from which the cells were harvested, DNA extracted, and confirmed as *Salmonella* based on molecular analysis [24].

### 2.5. Statistical Analysis

The experiment was a 6×6 factorial arrangement of treatments using four organic acid treatment concentrations, one untreated, a positive control, and six material contact surfaces. The mean log reductions of *Salmonella* for the treatments were subjected to two-way analysis of variance (ANOVA) using the GLIMMIX procedure of statistical analysis software SAS (version 9.3), and the treatment means were separated using Tukey’s posthoc test when the *F*-test of the ANOVA per treatment was significant at *p* < 0.05 [25].

## 3. Results

Logarithmic reductions in the *Salmonella* counts were observed on the contact surface materials due to treatment with the organic acid mixtures Activate DA and Activate US WD-MAX (Table 1).

**Figure 2:**
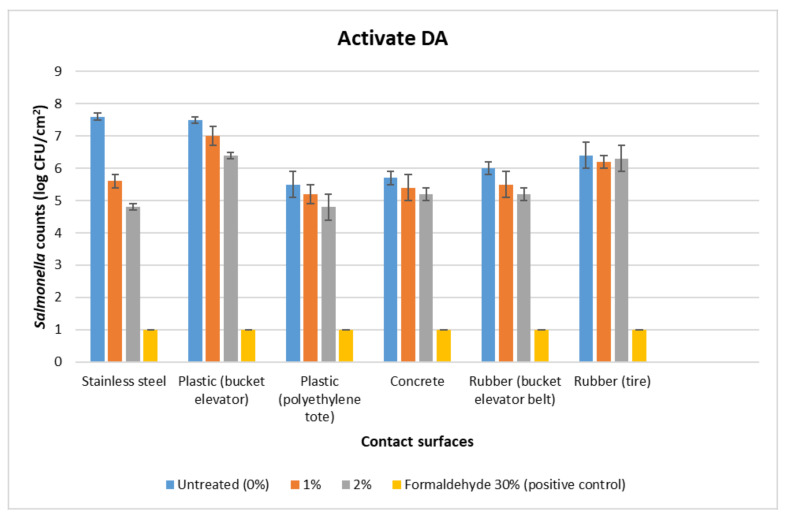
Mean logarithmic counts (log CFU/cm^2^) of *Salmonella* on food contact surfaces treated with organic acid mixture Activate DA at 1% and 2% concentrations in comparison to the untreated (0% organic acid) and positive control (30% formaldehyde). Limit of detection is 1 log CFU/mL for this study.

**Figure 3:**
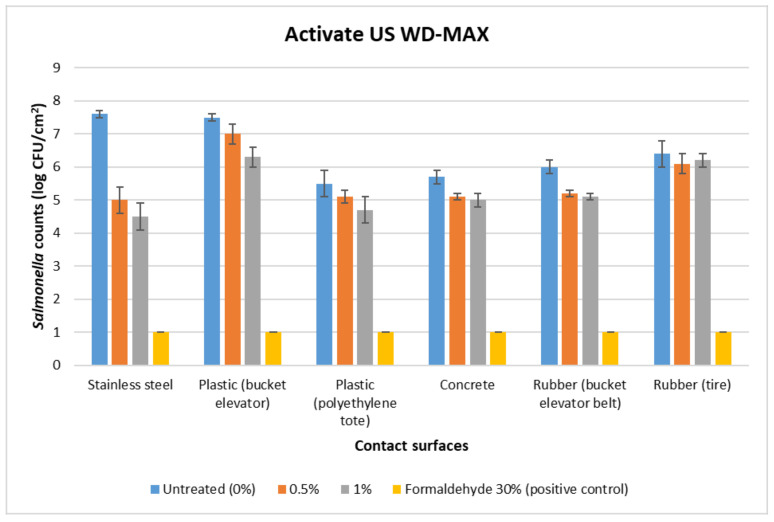
Mean logarithmic counts (log CFU/cm^2^) of *Salmonella* in food contact surfaces treated with organic acid mixture Activate US WD-MAX at 0.5% and 1% concentrations in comparison to the untreated (0% organic acid) and positive control (30% formaldehyde). Limit of detection is 1 log CFU/mL for this study.

The initial load of *Salmonella* in the inoculum was 8 log CFU/mL. For the untreated surfaces, the *Salmonella* counts were the highest on stainless steel at 7.6 log CFU/cm^2^, followed by plastic (bucket elevator), which had 7.5 log CFU/cm^2^ (Figure 2 and Figure 3). The *Salmonella* counts were the lowest on the plastic (tote) at 5.5 log CFU/cm^2^. The concrete and rubber (tire, belt) had intermediate counts (5.7–6.4 log CFU/cm^2^). The positive control (30% formaldehyde) showed the complete eradication of *Salmonella*.

For both Activate DA and Activate US WD-MAX organic acid treatments, plastic (bucket elevator) and rubber (tire) had the highest post-treatment *Salmonella* counts across all the concentrations tested (6–7 log CFU/cm^2^), and stainless steel and plastic (tote) had the lowest *Salmonella* recovered counts (4.5–5.6 log CFU/cm^2^) (Figure 2 and Figure 3).

The untreated surfaces showed log reductions in *Salmonella* in the range of 0.4–2.5 logs, even without organic acid treatment, due to differences in bacterial attachment and recovery from the various material surfaces (Table 1). When compared to the untreated control, the organic acid treatments showed greater reductions in *Salmonella* counts (*p* < 0.05). Across the various contact surface materials, Activate US WD-MAX at 0.5% had log reductions in the range of 1–3 logs, and at 1%, it had log reductions of 1.7–3.5 logs. Activate DA at 1% had log reductions of 1–2.8 logs, and at 2%, it had log reductions of 1.6–3.2 logs (Table 1).

For Activate US WD-MAX at 0.5%, the highest log reductions (3 logs) were observed for stainless steel, followed by plastic (tote) and concrete. Plastic (bucket elevator) had the lowest log reduction (1 log) of *Salmonella,* followed by rubber (tire). For Activate US WD-MAX at 1%, the highest log reduction of 3.5 logs was observed for stainless steel, followed by plastic (tote) and concrete. The lowest log reduction (1.7 logs) was observed for plastic (bucket elevator), followed by rubber (tire) (Table 1).

For 1% Activate DA, the highest log reductions (2.8 logs) were observed for plastic (tote), followed by concrete and rubber (belt), and the lowest log reduction (1 log) was observed for plastic (bucket elevator), followed by rubber (tire). For 2% Activate DA, the highest log reduction of 3.2 logs was observed for stainless steel and plastic (tote), and the lowest log reduction (1.6 logs) was observed for plastic (bucket elevator) and rubber (tire) (Table 1).

The differences between the treatment means within each treatment are reported in Table 1. There was no significant difference (*p* > 0.05) between the two organic acid treatments Activate DA and Activate US WD-MAX on the log reduction of *Salmonella* on the contact surfaces.

## 4. Discussion

The mitigation of *Salmonella* in food manufacturing facilities includes strategies like the minimization of pathogen entry, point-in-time mitigation, and the mitigation of postprocessing contamination [26]. Food ingredients can be potential vectors of pathogenic bacteria, and the contaminated ingredients can contaminate the facility equipment, leading to the cross-contamination of other products. Thermal food processing techniques, such as extrusion, can reduce and/or eliminate pathogens in foods [27,28]. However, postprocessing contamination with pathogens, including *Salmonella,* can occur during the manufacturing, storage, and transportation of finished food products through dust, air, or employee handling, which can lead to residual contamination in the finished product processing areas [29]. In order to prevent the spread of such biological hazards, food manufacturing facilities have traditionally relied on good manufacturing practices (GMPs) and on the implementation of hazard analysis and critical control point (HACCP) plans. GMPs rely on a facility design and layout that are amenable to effective physical cleaning, guidelines (for employee hygiene), and effective pest management to manage the prevention of biological hazards being introduced into the facility [30]. Many facilities rely on the removal of food/ingredient residues and dust to manage further contamination, although physical cleaning usually is not an effective sanitation method [9]. Huss et al. [9] described that highly intensive liquid sanitation and heat was required to completely rid an animal feed manufacturing facility of biological hazards. Therefore, it is safe to assume that food manufacturing equipment and contact surfaces may require substantial sanitization. The sanitization of surfaces can reduce postprocessing cross-contamination and can be applied throughout the facility to decontaminate equipment if undesirable micro-organisms like *Salmonella* have established themselves.

In this study, two organic acid mixtures, Activate DA (dry formula) and Activate US WD-MAX (wet formula), which are food-safe, were evaluated and were found to be effective in reducing *Salmonella* contamination on various surfaces, namely plastic (bucket elevator, tote bag), rubber (bucket elevator belt, automobile tire), stainless steel, and concrete. These material surfaces were chosen for evaluation as they encompass most of the different contact surfaces seen in an animal food manufacturing facility. For example, Davies and Wales [31] found that dust, spillage, and aggregated materials at all stages of the food manufacturing operation, including intake pits, ingredient silos, transfer augers, bucket elevators, weighing and mixing vessels, milling equipment, conditioners, pellet mills, coolers, finished product bins, and out-load gantries, could serve as vectors of pathogen contamination. The floors of such facilities are usually concrete, which can harbor pathogens; the products get transferred within the facility using plastic tote bags, which can get cross-contaminated with pathogens, and there are transport trucks that enter the facilities that can bring pathogens in through the automobile tires.

In this study, it was found that *Salmonella* counts were the highest (7.5–7.6 log CFU/cm^2^) on stainless steel and plastic (bucket elevator) when compared to the other surfaces tested (rubber, plastic from tote, and concrete) when no organic acid treatment was applied. These results are similar to that of the study by Muckey [26], who reported that stainless steel and plastic were the most challenging surfaces to sanitize in an animal feed manufacturing facility using liquid sanitizers. The reason could be because the elevator bucket plastic surfaces that we used in our experiment were ones that had received wear and tear from their use at the Hal Ross flour mill, KSU. When the surface of the plastic surface was not very smooth, it could harbor the growth of bacteria, and this aligns with practical situations wherein the bucket elevators in food manufacturing facilities undergo wear and tear, and when food residues collect in the boot pit or bottom, this can propagate the harboring and growth of pathogens like *Salmonella*. Similarly, stainless steel is known to support the pathogen growth of bacteria like *Salmonella,* which can form biofilms, as evaluated by previous research studies by Ronner et al. [32], Shen et al. [33], and Soni et al. [34]. This shows that the plastic from the bucket elevator and stainless steel can harbor *Salmonella* bacterial cells to a greater extent compared to other material surfaces.

The type of material surface had an effect (*p* < 0.05) on the log reductions obtained using the organic acid treatments (Table 1). Upon treatment with the wet organic mixture Activate US WD-MAX, the stainless steel and plastic (tote) surfaces had the highest log reductions in *Salmonella* (3–3.5 logs). This could be due to the smooth surface of stainless steel and plastic tote, which helped in exposing the bacterial cells to the organic acids greatly. Plastic (bucket elevator) and rubber (tire) had the lowest log reductions of *Salmonella* (1–1.7 logs) after treatment with Activate US WD-MAX. Rubber (tire) is a corrugated surface and can have minuscule pores and tears, and due to wear and tear, elevator bucket plastic does not have a very smooth surface. Rubber (elevator belt) surfaces also have cracks and pits that can further develop additional surfaces to harbor bacteria, and previous research has demonstrated that rubber surfaces can resist sanitation by showing increased growth of bacteria like *Listeria* and *Salmonella* [32]. These could be reasons why Activate US WD-MAX was the least effective on these surfaces. Almost similar log reduction results were observed for Activate DA dry formula treatment, where the lowest log reductions in *Salmonella* were observed for plastic (bucket elevator) and rubber (tire) (~1.6 logs). The highest reductions were observed for plastic (tote), stainless steel, and concrete (2.8–3.2 logs), probably due to their smooth surfaces, as explained earlier. In our experiment, we also found that stainless steel was more wettable with water than plastic (bucket elevator) and rubber (tire), which explains why the treatment with the organic acid mixtures was more effective on the stainless-steel surfaces than the others.

This study indicates that both Activate DA and US WD-MAX were effective at reducing *Salmonella* counts on food contact surfaces by 1–3.5 logs, with the higher concentrations tested (Activate US WD-MAX at 1% and DA at 2%) causing reductions of 1.6–3.5 logs. According to the United States Environmental Protection Agency (US-EPA), a sanitizer should demonstrate at least a 3-log reduction (99.9%) of the test micro-organisms (a Gram-positive and a Gram-negative organism) in 5 min on nonfood-contact surfaces (e.g., rubber tire) to be effective, while it should show at least a 5-log reduction (99.999%) in 30 s for food contact surfaces (e.g., stainless steel). In our study, about a 2.8–3.5 log reduction was achieved in the case of stainless steel, plastic (tote), and concrete due to treatment with the organic acid mixtures Activate DA and US WD-MAX at 2% and 1%, respectively.

There have been previous research studies that have evaluated the efficacy of natural/food-safe components, such as sanitizers, on food contact surfaces to mitigate pathogens, including *Salmonella* [34,35,36]. However, these sanitizers, being plant essential oils, were not economically feasible for application in a food manufacturing facility. Whereas, Activate DA and US WD-MAX were not as expensive as plant essential oils, and hence can be potentially feasible to apply as a sanitizer or antimicrobial in an industrial setting at optimum concentrations of 1–2%.

The mechanism of antibacterial activity of organic acids against Gram-negative bacteria like *Salmonella* had been described in previous research studies [37,38,39,40]. Organic acids in their undissociated and uncharged state are capable of bypassing bacterial cell membranes due to their lipophilic nature. Upon entering the more alkaline interior of a bacterium, the anion and proton from organic acids may have deleterious effects on the bacterium by increasing osmotic stress and disrupting important biomolecule synthesis, which finally causes bacterial death.

Activate DA, which is a dry, powdered formula, was evaluated in this study at 1% and 2% by dissolving it in water *w*/*w*. We did not evaluate it as a dry sanitizer due to the difficulty in its uniform application on the surface pieces, as a dry application would need a special applicator/equipment for uniform dusting at low or optimal amounts both in the laboratory and in a real food manufacturing facility. However, Muckey [26] reported that dry sanitizers have an advantage over wet sanitizers, as the wet sanitizers require rinsing with water and complete drying prior to later manufacturing in the facility. Muckey [26] evaluated sodium bisulfate (a dry acidulant) as a dry sanitizer on food contact surfaces and obtained a 2.7 log reduction in *Salmonella* on stainless steel. From this study, we, too, propose evaluating Activate DA as a dry sanitizer on food contact surfaces so that it could potentially be used in facilities handling dry-bulk systems. Muckey [26] also evaluated a commercial food-grade sanitizer and obtained a 1.9 log reduction on stainless steel. Compared to this, the food-grade organic acid mixtures tested in our study, Activate DA and Activate US WD-MAX, showed a higher log reduction of 2.8–3.5 logs on stainless steel against *Salmonella*.

It was reported in a previous study [23] that Activate DA and Activate US WD-MAX had effective antimicrobial activity when applied as a coating on pet food kibbles at 0.5–2%. This property of inclusion in these organic acid mixtures in the manufactured food can also act as a potential sanitizing agent for the food contact surfaces, as, for example, when the organic acid-treated food kibbles pass through a conveyor, they can have potential antimicrobial activity on the surface of the conveyor they come into contact with over an extended period of time. However, this method of antimicrobial activity warrants validation.

The corrosiveness of the organic acid mixtures evaluated in this experiment was not measured and was outside the scope of this experiment. However, this is an important aspect to consider when evaluating sanitizers, and so we propose testing for equipment corrosiveness using Activate DA and Activate US WD-MAX in future experiments. Additionally, higher concentrations (>2%) and treatment times (>15 min) of the organic acid mixtures could be evaluated in future studies to achieve higher log reductions in *Salmonella* on these surfaces, although factors like cost and application feasibility should also be considered.

## 5. Conclusions

In summary, surfaces and equipment in food manufacturing facilities can get contaminated with pathogens like *Salmonella* via postprocessing cross-contamination, with plastic and stainless-steel surfaces being more susceptible to harboring and sustaining the growth of *Salmonella*. The evaluation and selection of a sanitizer should consider microbial efficacy, the practicality of application, application time, impact on surface type for effectiveness and corrosiveness, and cost [41]. Prior to applying a sanitizer treatment to any surface, cleaning is necessary to reduce surface tension and remove organic material. Effective cleaning, which may require both physical cleaning and the use of cleaning solutions, removes biofilm formations, which then allows for the subsequent penetration and removal of vegetative bacteria via a sanitizer. Inadequate removal of organic matter during physical cleaning can provide adequate conditions for bacterial growth, increase cross-contamination, and reduce sanitizer efficacy [9]. From this study, it can be considered that Activate US WD-MAX at 1% and Activate DA at 2% can be effective sanitizers on food contact surfaces, including steel, rubber, and plastic. While this study yielded valuable data that represent a starting point for identifying Activate DA and Activate US WD-MAX as potentially effective sanitizers, additional research is warranted to determine the practical dosages and application methods based on cost and feasibility in an industrial setting.

## Figures and Tables

**Table 1:** Mean logarithmic reduction (log CFU/cm^2^) in *Salmonella* counts on pet food contact surfaces treated with organic acid mixtures Activate DA at 1% and 2% and Activate US WD-MAX at 0.5% and 1% concentrations in comparison to untreated (0% organic acid). The logarithmic reductions were calculated as the difference between the *Salmonella* counts in the inoculum (8 log CFU/mL) and on the contact surfaces (untreated and treated).

Food Contact Surface	*Salmonella* log Reduction (log CFU/cm^2^) (Mean ± SE) ^1,2,3^				
	Untreated	Activate US WD-MAX		Activate DA	
	0.0%	0.5%	1.0%	1.0%	2.0%
Stainless steel	0.4 ± 0.1 ^a,B^	3.0 ± 0.4 ^b,B^	3.5 ± 0.4 ^b,B^	2.4 ± 0.2 ^b,B^	3.2 ± 0.1 ^b,B^
Plastic (Bucket elevator)	0.5 ± 0.1 ^a,D^	1.0 ± 0.3 ^b,D^	1.7 ± 0.3 ^b,D^	1.0 ± 0.3 ^b,D^	1.6 ± 0.1 ^b,D^
Plastic (Tote)	2.5 ± 0.4 ^a,A^	2.9 ± 0.2 ^b,A^	3.3 ± 0.4 ^b,A^	2.8 ± 0.3 ^b,A^	3.2 ± 0.4 ^b,A^
Concrete	2.3 ± 0.2 ^a,AB^	2.9 ± 0.1 ^b,AB^	3.0 ± 0.2 ^b,AB^	2.6 ± 0.4 ^b,AB^	2.8 ± 0.2 ^b,AB^
Rubber (Belt)	2.0 ± 0.2 ^a,AB^	2.8 ± 0.1 ^b,AB^	2.9 ± 0.1 ^b,AB^	2.5 ± 0.4 ^b,AB^	2.8 ± 0.2 ^b,AB^
Rubber (Tire)	1.6 ± 0.4 ^a,C^	1.9 ± 0.3 ^b,C^	1.8 ± 0.2 ^b,C^	1.8 ± 0.2 ^b,C^	1.7 ± 0.4 ^b,C^

^1^ Each mean is based on *n* = 3 replications. ^2^ Means among the treatments across concentrations followed by different letters in lower case are significantly different (*p* < 0.05, Tukey’s test). ^3^ Within each treatment concentration, means among contact surfaces followed by different letters in upper case are significantly different (*p* < 0.05, Tukey’s test).

## Data Availability

Data will be provided upon reasonable request by author Aiswariya Deliephan.

## Supplementary Materials

The following supporting information can be downloaded at: https://www.mdpi.com/article/10.3390/foods12040874/s1, Figure S1: Plastic (bucket elevator); Figure S2: Rubber (bucket elevator belt); Figure S3: Rubber (automobile tire); Figure S4: Plastic (tote); Figure S5: Stainless Steel; Figure S6: Concrete.
