# Supplementary figures and images for: Mitigation of Salmonella on Food Contact Surfaces by Using Organic Acid Mixtures Containing 2-Hydroxy-4-(methylthio) Butanoic Acid (HMTBa)
Source: Foods. 2023 Feb 18;12(4):874. doi: 10.3390/foods12040874 (PMC9956140; doi:10.3390/foods12040874)

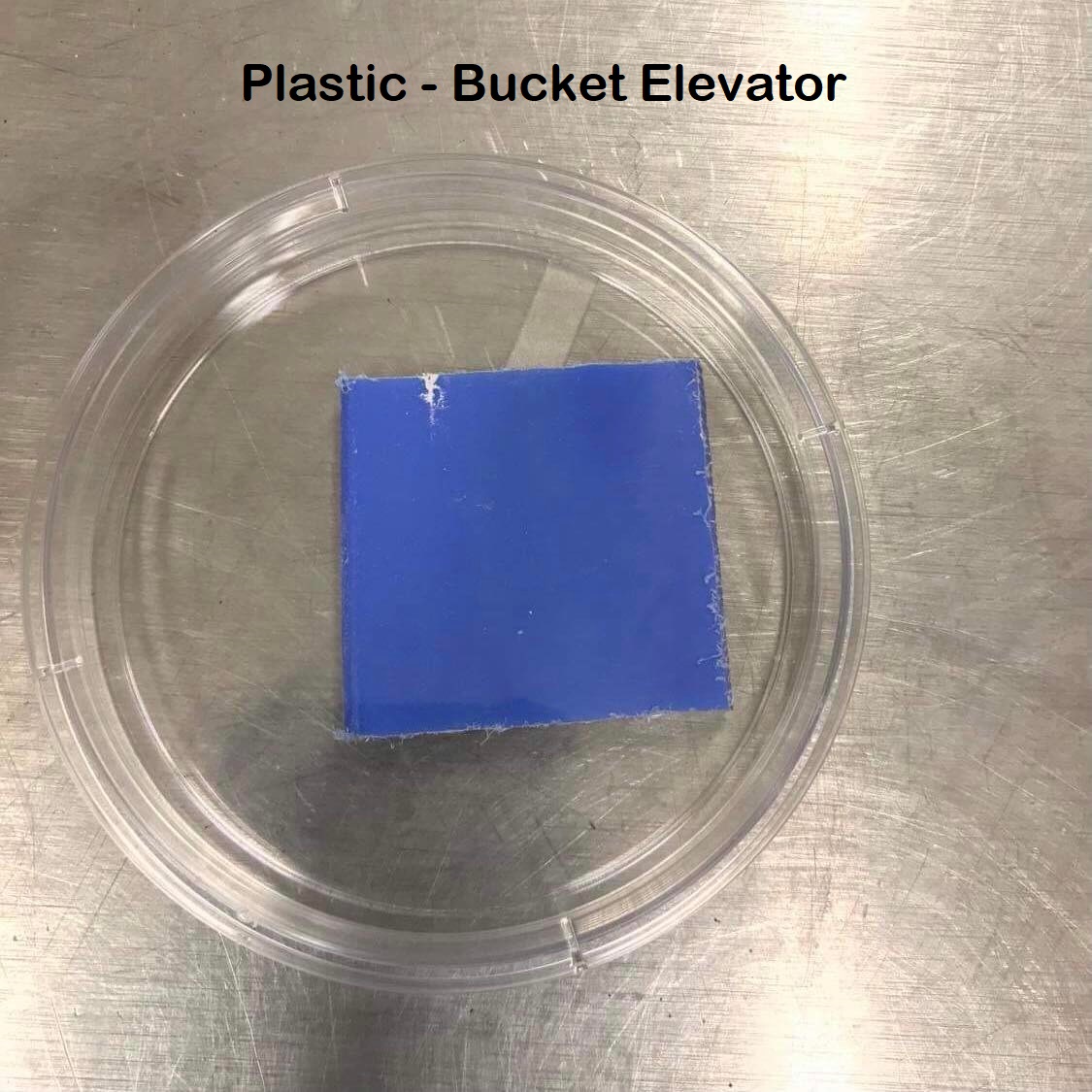

Supplement: Supplementary file 1 [file foods-12-00874-s001.zip › Figure S1-Plastic (bucket elevator).jpg]

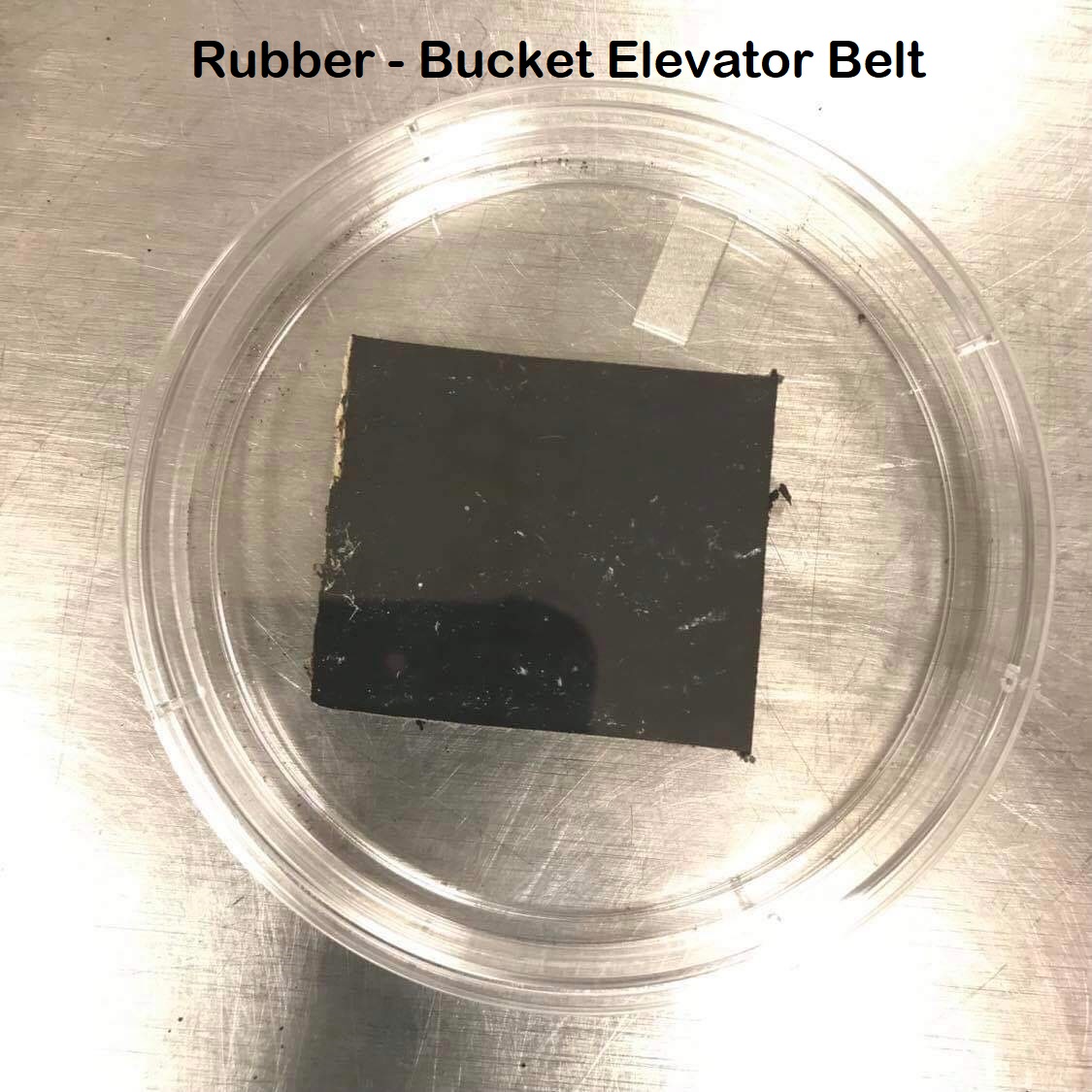

Supplement: Supplementary file 1 [file foods-12-00874-s001.zip › Figure S2-Rubber (bucket elevator belt).jpg]

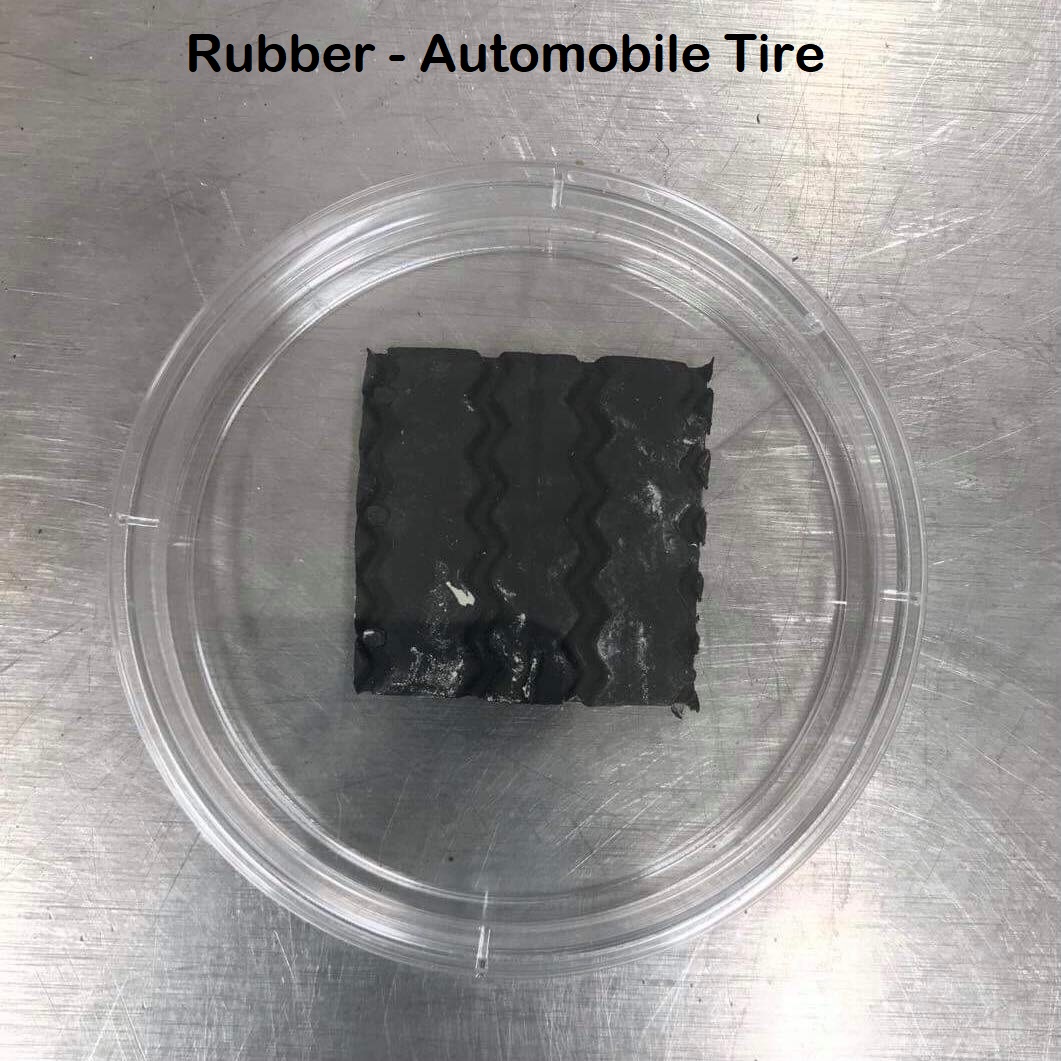

Supplement: Supplementary file 1 [file foods-12-00874-s001.zip › Figure S3-Rubber (automobile tire).jpg]

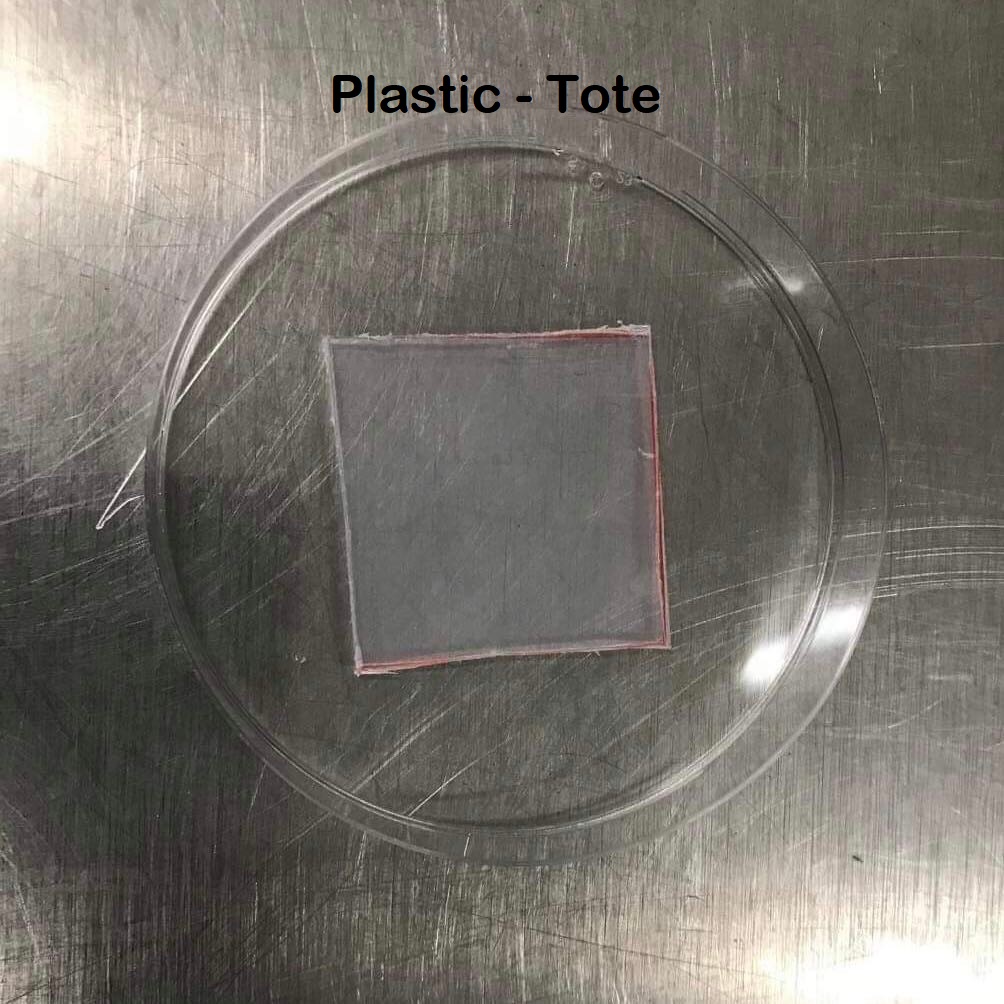

Supplement: Supplementary file 1 [file foods-12-00874-s001.zip › Figure S4-Plastic (tote).jpg]

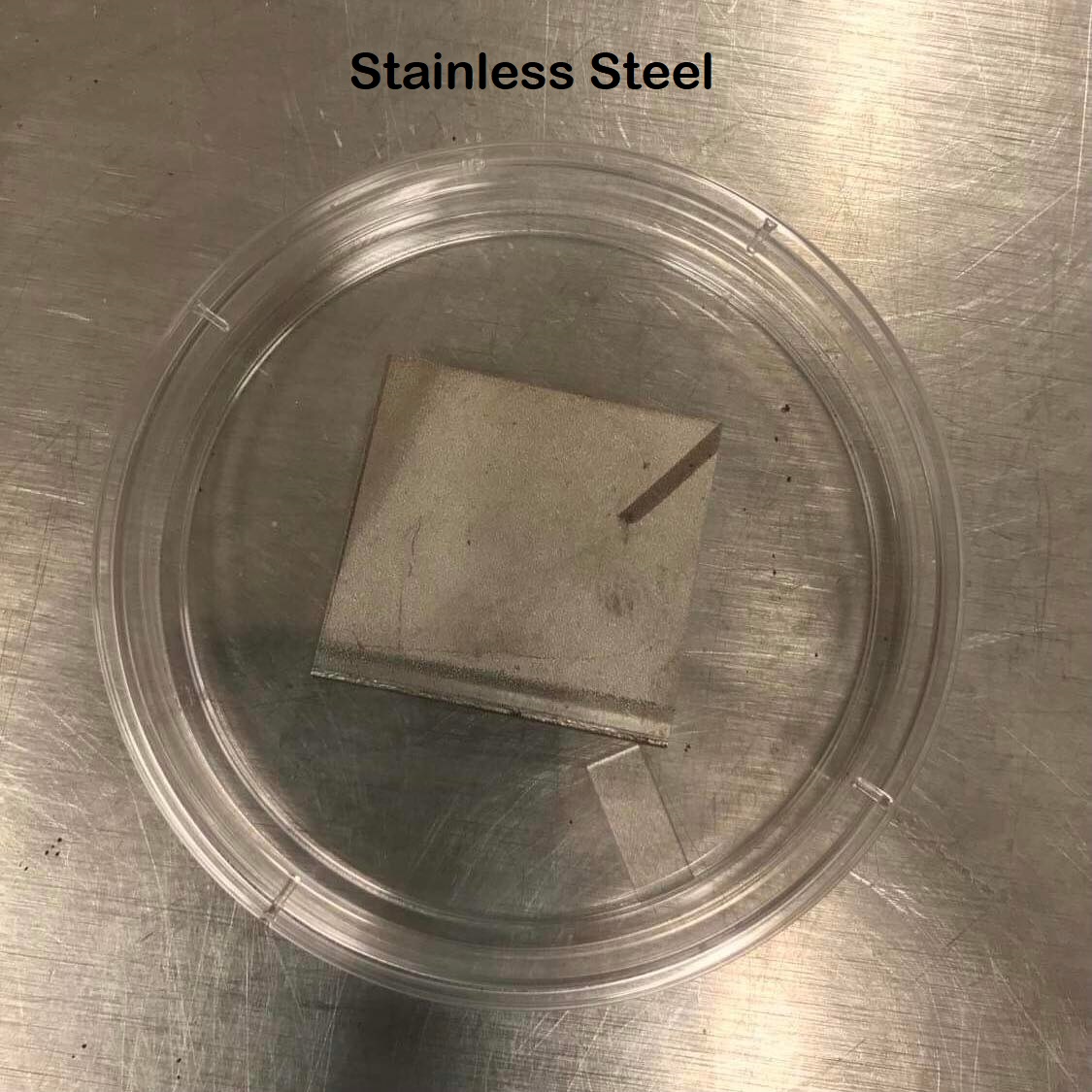

Supplement: Supplementary file 1 [file foods-12-00874-s001.zip › Figure S5-Stainless steel.jpg]

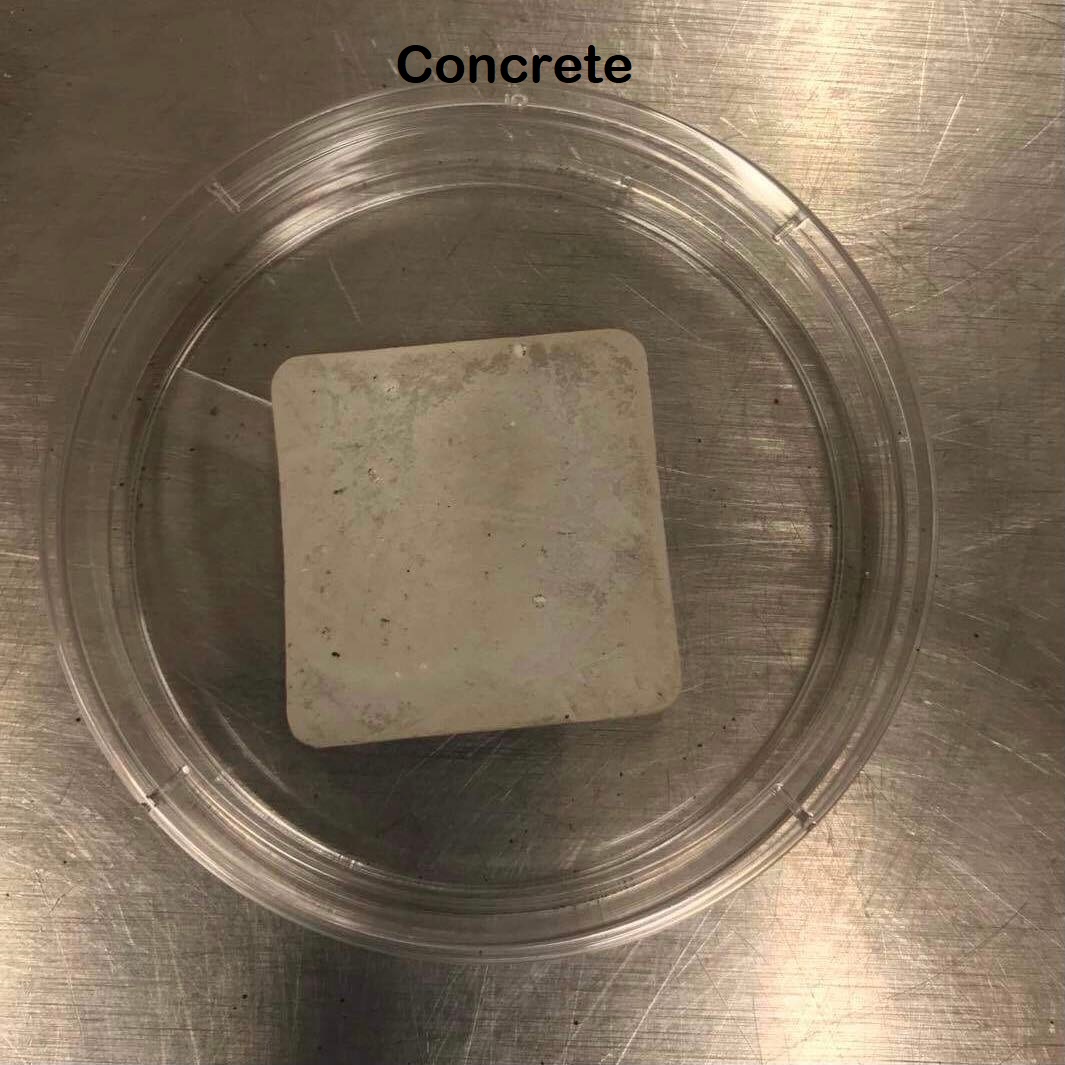

Supplement: Supplementary file 1 [file foods-12-00874-s001.zip › Figure S6-Concrete.jpg]
